# Predicting intracranial hemorrhage after traumatic brain injury in low and middle-income countries: A prognostic model based on a large, multi-center, international cohort

**DOI:** 10.1186/1471-227X-12-17

**Published:** 2012-11-19

**Authors:** Saleena Subaiya, Ian Roberts, Edward Komolafe, Pablo Perel

**Affiliations:** 1 Department of Emergency Medicine, New York Presbyterian, New York, NY, USA; 2 CRASH Trials Coordinating Center, The London School of Hygiene and Tropical Medicine, London, UK; 3 Department of Neurosurgery, Obafemi Awolowo University, Ife Ife, Nigeria

**Keywords:** Neurosurgery, Trauma, Developing countries, Intracranial hemorrhage, Traumatic brain injury

## Abstract

**Background:**

Traumatic brain injury (TBI) affects approximately 10 million people annually, of which intracranial hemorrhage is a devastating sequelae, occurring in one-third to half of cases. Patients in low and middle-income countries (LMIC) are twice as likely to die following TBI as compared to those in high-income countries. Diagnostic capabilities and treatment options for intracranial hemorrhage are limited in LMIC as there are fewer computed tomography (CT) scanners and neurosurgeons per patient as in high-income countries.

**Methods:**

The Medical Research Council CRASH-1 trial was utilized to build this model. The study cohort included all patients from LMIC who received a CT scan of the brain (n = 5669). Prognostic variables investigated included age, sex, time from injury to randomization, pupil reactivity, cause of injury, seizure and the presence of major extracranial injury.

**Results:**

There were five predictors that were included in the final model; age, Glasgow Coma Scale, pupil reactivity, the presence of a major extracranial injury and time from injury to presentation. The model demonstrated good discrimination and excellent calibration (c-statistic 0.71). A simplified risk score was created for clinical settings to estimate the percentage risk of intracranial hemorrhage among TBI patients.

**Conclusion:**

Simple prognostic models can be used in LMIC to estimate the risk of intracranial hemorrhage among TBI patients. Combined with clinical judgment this may facilitate risk stratification, rapid transfer to higher levels of care and treatment in resource-poor settings.

## Background

Traumatic brain injury (TBI) is a leading cause of death and disability worldwide, affecting approximately 10 million people annually according to the World Health Organization. This burden disproportionately affects low and middle-income countries (LMIC), with annual TBI-related incidence rates of 150–170 per 100,000 people as compared to the global rate of 106 per 100,000 [1]. Those in LMIC are twice as likely to die following severe TBI as compared to those in high-income countries [2].

Intracranial hemorrhage is a frequent and devastating sequelae of TBI, occurring between one-third to a half of cases [3,4]. Intracranial hemorrhage is the leading cause of death in lethally injured trauma patients accounting for 40-50% of fatalities [5] and results in a significant amount of long-term disability [6].

It has been suggested that organized emergency response systems and prompt transfer to trauma centers improve TBI patient morbidity and mortality [7]. An important adjunct to this is the availability of computed tomography (CT) scanners and neurosurgeons, with rapid surgical intervention resulting in a reduction in deaths [8]. CT scanning is the imaging modality of choice in the identification of intracranial hemorrhage due to its speed and diagnostic capabilities, however, there is only one scanner per 3.5 million people in low-income countries versus one per 64,900 in high-income countries [9]. There are also fewer neurosurgeons per patient, with one neurosurgeon per three million patients in Sub-Saharan Africa as compared to one per 20,000 in Europe [10]. Scarce resources in LMIC compounded with the increased burden of TBI make this a pressing public health issue.

Prognostic modeling provides a unique opportunity to aid clinical judgment and diagnostic ability, as they combine readily available patient data to predict the possibility of an outcome of interest [11,12]. The utility of these models in regards to TBI have shown to influence patient, next-of-kin and physician decision-making [13,14]. Additionally, they have been demonstrated to be more accurate than a physician’s own predictive capabilities [13]. This can have a particularly important role in LMIC as there is a lack of specialty training in trauma among the healthcare workforce and diagnostic capabilities are limited [12,15]. The understanding and application of prognosis can be utilized in this setting to risk-stratify patients, and assist both care providers and family members with decisions to transfer patients to higher levels of care.

However, there is a paucity of prognostic models on TBI in LMIC, and no models currently exist that predict the risk of intracranial hemorrhage in this setting. The models that do exist suffer from multiple methodological flaws, including small sample sizes from a single center, inappropriate validation methods, and a lack of calibration or discrimination [16]. This highlights the necessity of new research to create accurate TBI prognostic modeling to aid clinicians with outcome prediction, as single factors do not have sufficient predictive value [17].

The Medical Research Council CRASH-1 (corticosteroid randomization after significant head injury) trial is the largest randomized controlled trial to date conducted in patients with TBI from 2005 [18,19]. The trial prospectively included patients within eight hours of injury, standardised their definitions of risk factors, and obtained CT scans of the head in over 75% of their patients. This allows for a large sample size to ensure high precision and valid prediction. Additionally, high recruitment of patients from LMIC allows for the identification of prognostic factors that are relevant to these settings. The results of this study demonstrated an association with corticosteroids and increased mortality of TBI patients. Prognostic models have been developed from this data to evaluate morbidity and mortality among TBI patients and have been externally validated in several settings; however, prediction of intracranial hemorrhage was not done [3,20,21].

The purpose of our study is to identify readily available risk factors for intracranial hemorrhage, and build a clinically useful prognostic model for intracranial hemorrhage among TBI patients in LMIC that can be used by those without specialty training in neurosurgery or trauma.

## Methods

### Selection of participants

The study cohort was composed of all patients enrolled in the CRASH-1 trial from LMIC who received a CT scan of the brain. Adults aged 16 or older with TBI defined as any head injury with impaired consciousness (Glasgow coma score of 14 or less), and who were within eight hours of injury were eligible for inclusion in this trial [22]. The level of income of the country was defined as low, middle, or high- income countries as defined by the World Bank where middle was comprised with both low-middle and high-middle income [23]. Patients from low and middle-income countries were included in our analysis (Table 1).

**Table 1:** Low and middle-income countries as defined by the World Bank included in the CRASH trial

**Countries**	**Number of patients enrolled**
Albania	41
Argentina	359
Brazil	119
Chile	3
China	87
Colombia	832
Costa Rica	20
Ecuador	258
Egypt	775
Georgia	56
Ghana	7
India	973
Indonesia	238
Iran	233
Ivory Coast	3
Kenya	2
Malaysia	176
Mexico	17
Nigeria	180
Pakistan	17
Panama	7
Paraguay	10
Peru	8
Romania	319
Serbia	23
South Africa	366
Sri Lanka	132
Thailand	579
Tunisia	63
Turkey	2
Uganda	43
Vietnam	2

### Outcomes

CT scan diagnosis of intracranial hemorrhage was defined as the presence of subarachnoid hemorrhage, petechial hemorrhages, obliteration of third ventricle or basal cisterns, mid-line shift, evacuated hematomas and non-evacuated hematomas. These were dichotomized to include all those diagnosed by CT-scan to have intracranial hemorrhage, and those with a CT-scan who did not. Patients were administered a CT-scan based on the clinical judgment of their physician.

### Prognostic variables

We considered age, sex, Glasgow Coma Scale (GCS), time from injury to randomization, pupil reactivity, cause of injury, seizure and whether the patient had sustained a major extracranial injury. These variables were all pre- and post- injury factors included in the CRASH-1 trial excluding hematemesis or melena, the presence of a wound infection, or pneumonia. These were selected for inclusion in our study as prior research has demonstrated a relationship between these variables and the presence of intracranial hemorrhage [24,25]. The analysis was adjusted for randomization to corticosteroids as this was related to increased mortality within the trial. We also assessed for the presence of interaction between treatment and potential prognostic factors as well as between prognostic factors for our model.

### Analyses

All statistical analyses were conducted using STATA 10 (College Station, TX, USA). Univariate analysis was conducting using logistic regression modeling using the maximum likelihood theory to evaluate the relationship between prognostic variables and outcomes. We quantified each variable’s predictive contribution by its z score (the model coefficient divided by its standard error). We graphically explored the relationship between age and intracranial hemorrhage, and GCS and intracranial hemorrhage to assess for linearity.

### Prognostic models

The final model in multivariate analyses was built using backwards elimination, where all variables were initially included [26]. Variables were selected for elimination using a p-value of 0.05, whereby a series of likelihood ratio tests with a p-value of <0.10 were utilized to determine which variables were kept in the final model. We explored for interaction between treatment and all other variables included in the final model using the likelihood ratio test. Ninety-five percent confidence intervals (CI) and p-values were calculated for all statistical tests of association. As there were few missing data, a complete case analysis was performed.

### Performance of the model

The performance of the model was assessed through calibration and discrimination. Calibration was evaluated graphically by plotting the observed proportion of events against predicted risks for 10 risk groups of equal size, as well as statistically with the Hosmer-Lemeshow test. Discrimination of the model was assessed using the c-index.

### Internal validation

The internal validity of the final model was assessed by the bootstrap re-sampling technique. Regression models were estimated in 50 models. For each of 50 bootstrap samples we refitted and tested the models on the original sample to obtain an estimate of predictive accuracy corrected for bias.

### Risk score estimation

A clinical score was created using regression coefficients and a percentage risk calculated from these coefficients with an absolute risk equation. The absolute risk is expressed as a range of percentages for a given clinical score to facilitate its use in an emergency setting where the ability to do complex calculations may be limited.

### Ethics approval

As this was a secondary retrospective analysis of the CRASH-1 trial and there were no patient identifiers utilized, there was no additional IRB approval that was obtained for conduction of this study. All MRC CRASH collaborators obtained local ethics and/or research committee approval for the original CRASH-1 trial.

## Results

### General characteristics of study subjects

Descriptive characteristics of study subjects are displayed in Table 2. A total of 5,669 TBI patients underwent a CT scan in low- and middle-income countries, and 3917 (69.1%) were diagnosed with an intracranial hemorrhage. Among patients with intracranial hemorrhage, subarachnoid hemorrhage was present in 1900 (48.5%), petechial hemorrhage in 1629 (41.6%), hematomas not requiring evacuation in 1550 (39.6%) and hematomas requiring evacuation in 808 (20.6%).

**Table 2:** Descriptive characteristics of study population

**Variables**	**n**	**n ICH**	**% ICH**
**Age (years)**	5669	3917	69
> = 19	716	493	69
20-29	1747	1147	66
30-39	1187	819	69
40-49	863	597	69
50-59	580	412	71
60-69	305	236	77
70-79	201	157	78
>=80	70	56	80
	p^1^ < 0.001		
**Gender**	5669	3917	69
Male	4723	3296	70
Female	946	621	66
	p = 0.012		
**Time since injury (hour)**	5669	3917	69
<=1	1049	623	59
1- ≤3	1677	1121	67
>3	2943	2173	74
	p < 0.001		
**GCS**	5669	3917	69
Mild (13–14)	1294	641	50
Moderate (9–12)	1825	1189	65
Severe (3–8)	2550	2087	82
	p < 0.001		
**Pupil Reactivity**	5669	3917	69
Both reactive	4741	3102	65
One or both unreactive	928	815	88
	p < 0.001		
**Major Extracranial Injury**	5643	3897	69
None	4281	2961	69
Yes	1362	936	69
	p = 0.758		
**Type of injury**	5654	3909	69
Road traffic accident	4144	2875	68
Fall >2 metres	615	430	70
Other mechanism	895	604	68
	p = 0.998		
**Seizure**	5646	3900	69
No	5216	3603	69
Yes	430	297	69
	p = 0.998		
**Death at Two Weeks**	5669	3917	69
Alive	4386	2793	64
Dead	1283	1124	88
	p < 0.001		
**Death at Six Months**	5443	3762	69
Alive	3890	2410	62
Dead	1553	1352	87
	p < 0.001		

^1^p values represent significance testing for univariate odds ratios.

There was an increased frequency of intracranial hemorrhage with increasing age. Males were more likely than females to have an intracranial hemorrhage. The risk of intracranial hemorrhage increased with increasing time from injury to presentation. The presence of an intracranial hemorrhage was associated with death at both two weeks (*x*2 = 266.1, df = 1, p < 0.001), and at 6 months (*x*2 = 327.7, df = 1, p < 0.001).

The relationship between ten-year age categories and log odds of intracranial hemorrhage was linear, and therefore analysed as an ordered categorical variable. The relationship between GCS and log odds of intracranial hemorrhage was closely linear, and was therefore analysed as a continuous variable in multivariable analysis.

### Multivariable predictive models

There were five predictors that were included in the final model: age, GCS, pupil reactivity, the presence of a major extracranial injury and time from injury to presentation (Table 3). GCS was the strongest predictor, followed by time from injury to presentation, and age. The presence of a major extra-cranial injury was associated with a reduction in the risk of having an intracranial hemorrhage (ICH).

**Table 3:** Multivariable predictive model

**Exposure variables**	**Low and middle income countries**	
	**Adjusted odds ratio (95% CI**^**1**^**), z-score**	**p-value**^**2**^
Age^3^	1.10 (1.06, 1.15), 5.19	<0.001
Glasgow Coma Scale^4^	1.21 (1.18, 1.23), 17.46	<0.001
One or both pupils unreactive	1.81 (1.44, 2.26), 5.14	<0.001
Major extracranial injury	0.78 (0.68, 0.90), -3.35	0.001
Time since injury^5^	1.38 (1.27, 1.48), 8.19	<0.001
c-index: 0.71		

^1^ Ninety-five percent confidence interval.

^2^ Wald Test p-value for association between variables and outcome.

^3^ OR for a 10-year increase in age from a baseline group of <16.

^4^ OR for a one-unit decrease in GCS from a baseline of 14.

^5^ OR for a categorical increase in hours of injury until randomization (baseline <1 hr, 1- ≤3 hrs, >3 hrs).

#### Performance of the model

**Figure 1:**
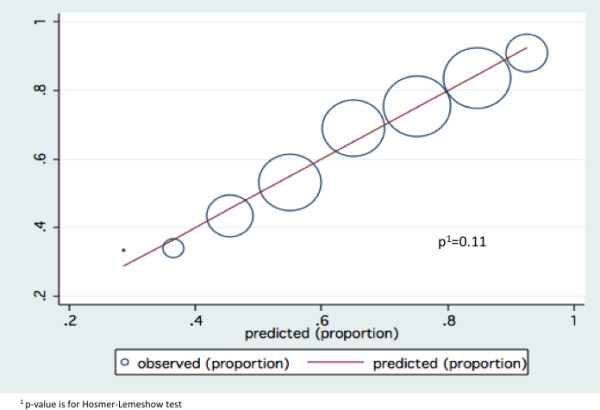
Calibration of final model.

The model showed good discrimination, with a c-statistics of 0.71. It demonstrated good calibration graphically and after evaluation with the Hosmer-Lemeshow test (Figure 1).

#### Internal validation

We did not find evidence of a significant overoptimism in our model development. The overoptimism for the c-statistic with the bootstrapping procedure was 0.15%.

#### Clinical risk score

Individual risk scores can be calculated from Table 4 and are associated with a corresponding risk percentage (Table 5). For example, the risk of intracranial hemorrhage in a 55-year-old TBI patient with a GCS score of 12, reactive pupils, no major extracranial injury who presents 3 hours after injury would have a calculated risk score of 14 which corresponds with an 60- < 65% risk of intracranial hemorrhage.

**Table 4:** Estimation of the risk score of intracranial hemorrhage

**Variable**	**Risk score**^**1**^
**Age (years)**	
> = 19	0
20-29	1
30-39	2
40-49	3
50-59	4
60-69	5
70-79	6
> = 80	7
**GCS**	
14	0
13	2
12	4
11	6
10	8
9	10
8	12
7	14
6	16
5	18
4	20
3	22
**Pupil Reactivity**	
Both reactive	0
One or both unreactive	6
**Major Extracranial Injury**	
None	0
Yes	−3
**Time since injury (hour)**	
<=1	0
1- ≤3	3
>3	6

^1^ Risk Score estimated from regression coefficients stratified by prognostic variable. Values have been rounded up to nearest whole number to facilitate operator use.

**Table 5:** Percentage risk of intracranial hemorrhage according to the risk score

**Risk score**	**Percentage risk**^**1,2**^
−3 - < 0	25- < 30
0 - < 3	30- < 35
3 - < 5	35- < 40
5 - < 7	40- < 45
7 - < 9	45- < 50
9 - < 11	50- < 60
11 - < 15	60- < 65
15 - < 17	65- < 70
17 - < 20	70- < 75
20 - < 23	75- < 80
23 - < 26	80- < 85
26 - < 31	85- < 90
31- < 39	90- < 95

^1^Percentage risk calculated from risk score utilizing the following equation: Percentage risk = 100 * (e^(0.1*score)^/(1+e^(0.1*score)^)).

^2^Percentage risk <50% has an margin of error of 1–1.5% points secondary to rounding of risk score.

## Discussion

We have developed a prognostic model utilizing readily available clinical data to predict the risk of intracranial hemorrhage in TBI patients from LMIC. The model has demonstrated good discrimination, excellent calibration and has been internally validated.

Advanced age, GCS, pupil reactivity, the presence of a major extracranial injury and time from injury to presentation were all found to be predictors for intracranial hemorrhage (ICH). GCS demonstrated a linear relationship with increased risk for intracranial hemorrhage, except for those with a calculated score of three. This could be attributed to those patients that have been sedated and intubated prior to recording of GCS, as these are given a score of three by default [27]. A linear relationship between advanced age and increased risk of poor outcome after TBI has been documented previously and was demonstrated in our study [24]. The increasing risk of hemorrhage with increasing time from injury to presentation may reflect the fact that slower bleeds are more likely to be detected at a later scan and could have been missed in early imaging. This can also be attributed to prolong extrication times, which has been demonstrated to be associated with major injury [25]. Additionally the possibility of bias must be considered, as patients referred for more serious injury may be more likely to present with a bleed. Also a change in neurological status or development of new clinical symptoms may prompt patients to seek delayed care after injury.

This study has limitations. In order to explore the generalisability of a prognostic model to a similar patient population within a different setting, external validation is necessary [28]. However, we did not have access to data that contains the patient population and variables included in this study, so external validation was not possible. Another limitation is the consideration of all intracranial hemorrhage together as variability in prognostic factors may exist depending on type of bleed. The use of CT scanning to diagnose intracranial hemorrhage across different centers is subject to interobserver variability however, because this potential measurement error is unrelated to prognostic factors the estimate of the prognostic factor remains unbiased, although potentially imprecise [29]. The exclusion of patients who did not have a CT scan is an additional limitation that may cause potential selection bias in our sample.

The strength’s of this study is the use of prospective, standardised data collection on prognostic variables, and a well-defined patient cohort with few losses to follow-up. Additionally, this is the largest sample of patients from LMIC with TBI to derive a prognostic model to our knowledge.

## Conclusion

This is the first study of its kind, to our knowledge, to provide a risk stratification of intracranial hemorrhage among TBI patients involving multiple prognostic variables. While other studies have evaluated prognostic variables to triage and treat trauma patients such as the New Orleans Criteria, The Canadian Head CT Rule, and the Trauma Score and Injury Severity Score, no risk score has been designed to specifically evaluate the risk of intracranial hemorrhage in TBI patients from LMIC [30-34]. The scores that do exist in this setting focus on morbidity and mortality after head injury and all suffer from methodological limitations [17].

Prognostic factors in TBI are often used within the context of clinical judgment and radiographic evidence to diagnose intracranial hemorrhage in patients. However, the utility of a single prognostic variable is limited and a combination of variables into a prognostic model could be a more useful clinical tool. While a prognostic model should never replace clinical judgment, it can be used in conjunction with professional knowledge to inform decision-making. Previous studies have demonstrated that prognostic modeling in TBI can be used to accurately access long-term outcomes [20]. Within LMIC this can be useful for diagnosis, referral, and treatment. However, although a prognostic model could help the decision making process and ensure a more rational use of limited resources, increase in TBI related resources (CT scan and neurosurgeons) in this setting is paramount to prevent long-term disability and mortality.

In summary, this model within this population demonstrated good performance; however, future research utilizing a prospective cohort design to perform external validation is needed. Further investigations should assess if the application of this risk score in a low-income settings would improve patients’ outcomes. While it would be worthwhile to determine a risk score for patients who had a neurosurgical intervention, inherent bias may flaw these studies, as physicians may be influenced to operate based on variables included in the model.

## Abbreviations

TBI: Traumatic brain injury; ICH: Intracranial hemorrhage; LMIC: Low and middle-income countries; CT: Computed tomography; GCS: Glasgow coma scale.

There was no grant funding or other financial support involved in this study. The original founders of the CRASH trial had no role in this study design, data collection and analysis, decision to publish, or preparation of the manuscript. The original funding for the CRASH-1 trial was obtained from the UK Medical Research Council.

## Competing interests

There are no financial, personal or professional interests that could be construed to have Influenced this paper.

## Authors’ contributions

SS, PP, and IR conceived the study. SS and PP created the statistical analysis plan and analyzed the data. EK provided key insight in creating an accessible and user-friendly risk score. SS drafted the manuscript, and all authors contributed substantially to its revision.

## Pre-publication history

The pre-publication history for this paper can be accessed here:

http://www.biomedcentral.com/1471-227X/12/17/prepub
